# Recent advances in understanding glucose transport and glucose disposal

**DOI:** 10.12688/f1000research.22237.1

**Published:** 2020-06-24

**Authors:** Ann Louise Olson, Kenneth Humphries

**Affiliations:** 1 Biochemistry & Molecular Biology, University of Oklahoma Health Sciences Center, Oklahoma City, OK, USA; 2 Aging & Metabolism Research Program, Oklahoma Medical Research Foundation, Oklahoma City, OK, USA

**Keywords:** glucose transport, glucose disposal, insulin resistance

## Abstract

Deficient glucose transport and glucose disposal are key pathologies leading to impaired glucose tolerance and risk of type 2 diabetes.  The cloning and identification of the family of facilitative glucose transporters have helped to identify that underlying mechanisms behind impaired glucose disposal, particularly in muscle and adipose tissue.  There is much more than just transporter protein concentration that is needed to regulate whole body glucose uptake and disposal.  The purpose of this review is to discuss recent findings in whole body glucose disposal.  We hypothesize that impaired glucose uptake and disposal is a consequence of mismatched energy input and energy output.  Decreasing the former while increasing the latter is key to normalizing glucose homeostasis.

## Introduction

One of the central metabolic features of type 2 diabetes in humans is decreased glucose disposal. This process is rate limited by the capacity for glucose transport into the peripheral and splanchnic tissues. The prevailing hypothesis for limited glucose transport and disposal in type 2 diabetes has centered on the function of the insulin-dependent glucose transporter GLUT4. This transporter, expressed at the highest levels in adipose tissue, skeletal muscle, and cardiac muscle, is recruited to the cell surface when activated by rising insulin levels following consumption of a meal. This hypothesis is supported in large part by studies performed using whole animal and tissue-specific transgenic manipulation of GLUT4 expression and *in vitro* studies of insulin-dependent glucose uptake in patients with type 2 diabetes ^1^. While it is clear that both insulin-dependent GLUT4 trafficking and total cellular GLUT4 pool size play a critical role in glucose disposal, other glucose transporters and other factors influence glucose uptake in tissues including interstitial glucose concentration and intracellular glucose metabolism. The contributions of transporter pool size, rate of glucose delivery to the tissues, competition with other energy substrates, and ATP turnover are the key issues that must be resolved to understand how to effectively prevent and treat type 2 diabetes.

## Regulation of glucose uptake by interstitial glucose concentration and blood flow

In human subjects with prediabetes and type 2 diabetes, impaired glucose uptake in skeletal muscle, visceral adipose tissue, and the brain appear to be the best predictors of insulin resistance ^2^. Glucose uptake in these tissues occurs by facilitative diffusion. Because glucose is rapidly phosphorylated once it enters the cell, the rate of glucose transport is dictated by the extracellular glucose concentration as well as the number of transport proteins found on the cell surface. In the case of visceral adipose tissue and skeletal muscle, the extracellular (or interstitial) glucose concentration is dictated by blood flow into the tissue. It has long been known that insulin action increases vasodilation, presumably to deliver nutrients and insulin to tissues (for a recent review, see 3). Unknown is the relative contribution of changes in cell surface transporter number and changes in interstitial glucose concentration to glucose uptake in tissues. To answer this question, McConell *et al*. sought to estimate insulin-dependent muscle glucose uptake in human subjects at rest and after a bout of exercise with and without modulation of blood flow to the tissue ^4^. In this study, subjects performed a one-legged knee extensor exercise so that glucose uptake could be measured in the exercised leg and compared to a parallel biopsy taken from the rested leg. Based on previous studies, the authors anticipated at least a twofold increase in insulin-dependent glucose uptake based on insulin-dependent translocation of GLUT4 to the cell surface. Instead, the increase in glucose uptake following submaximal euglycemic/hyperinsulinemic clamp was 17-fold in the rested leg and 36-fold in the exercised leg. The apparent twofold increase in glucose uptake in the exercised leg might be accounted for by the exercise-dependent increase in GLUT4 translocation, thought to be additive with insulin. To determine if muscle perfusion, a means of replenishing interstitial glucose, modulated glucose uptake, the authors infused NG-monomethyl L-arginine acetate (L-NMMA) into the femoral artery during the clamp to constrict the vasculature. Indeed, restriction of blood flow reduced glucose uptake. These data point out that both insulin-dependent and exercise-dependent increases in glucose uptake are regulated by both cell permeability and interstitial glucose concentration. The key question is whether glucose disposal in people with prediabetes or type 2 diabetes is limited exclusively by impairment in insulin-dependent GLUT4 translocation or insulin-dependent tissue perfusion. A partial answer to this question is found in the work of Henstridge *et al.*, where overweight and obese men with type 2 diabetes were given a nitric oxide (NO) donor, sodium nitroprusside, or verapamil to increase leg blood flow. While both drugs increased leg blood flow, only sodium nitroprusside increased glucose uptake in the treated muscle ^5^. The authors interpret this data to mean that increased blood flow alone is not sufficient to increase glucose uptake when membrane permeability (i.e. GLUT4 plasma membrane levels) is low. Furthermore, the authors raised the possibility that NO signaling was responsible for exercise-dependent GLUT4 translocation, a finding that has since been supported in both cultured myotubes and mouse skeletal muscle ^6, 7^.

In contrast to the human study by McConell *et al*., inhibition of blood flow by vasoconstriction in conscious, sedentary mice resulted in an increased transendothelial insulin efflux and, in turn, increased glucose clearance ^8^. The authors of this study demonstrated that vasoconstriction via inhibition of NO synthase (NOS) resulted in increased glucose clearance in response to an insulin tolerance test. While the results of these two studies are conflicting, it is important to note that the McConell study utilized a euglycemic/hyperinsulinemic clamp protocol that measures steady-state insulin action. The Williams study used an insulin bolus, which better represents insulin kinetics. It is likely that the initial increase in transendothelial insulin efflux increased glucose uptake in the first 15 minutes of NOS inhibition, compensating for decreased perfusion of the tissues.

## Balance between lipid metabolism and glucose disposal

A predisposing factor for developing insulin-resistant glucose transport is obesity and the lipid overload associated with that condition. Simply put, a high-lipid environment results in decreased glucose disposal. This line of thinking led to the notion that inhibiting fatty acid oxidation would increase glucose oxidation and glucose disposal, thus curing insulin resistance. This substrate competition model has been tested in mice using pharmacologic inhibition of carnitine palmitoyltransferase-1 (CPT-1) activity ^9^. Mice were fed a 45% fat diet for 6 days to increase fatty acid availability and then given a single injection of etomoxir to inhibit CPT-1. Within 4 hours, plasma glucose levels decreased and glucose oxidation in peripheral tissues increased. The authors observed that free fatty acid levels became elevated with the acute etomoxir treatment, and they became concerned that the lipidemic environment would be enhanced with long-term treatment. This prediction proved to be correct. Prolonged inhibition of fatty acid oxidation led to decreased glycemic control, hepatic insulin resistance, and fatty liver. Thus, the substrate competition hypothesis failed to provide a useful therapeutic hypothesis for the treatment of insulin resistance. More importantly, the prolonged inhibition of fatty acid oxidation led to decreased glucose clearance, further supporting the idea that a high-lipid environment can impair glucose transport and glucose disposal. While long-term clinical trials of etomoxir in human subjects have not been carried out, a short-term clinical trial showed that treatment of subjects with type 2 diabetes with etomoxir for 3 days increased glucose oxidative metabolism under basal conditions but had no effect under hyperinsulinemic clamp conditions ^10^. This study suggested that inhibition of fatty acid oxidation would not improve insulin sensitivity in patients with type 2 diabetes.

If increasing glucose oxidation does not improve metabolic homeostasis in a high-lipid environment, can increased lipid oxidation solve the problem? This question was approached by supplementing carnitine in individuals with impaired glucose tolerance and normal glucose tolerance ^11^. Carnitine supplementation clearly increased plasma carnitine levels and synthesis of acetylcarnitine levels in the muscle, indicating that carnitine concentration is rate limiting for fatty acid oxidation. These carnitine-dependent increases in fatty acid metabolism did not improve insulin sensitivity or change carbohydrate oxidation. Thus, substrate switching alone is not sufficient to improve glucose transport and glucose disposal in glucose-intolerant volunteers.

## Regulation of glucose disposal by increased energy expenditure

Some light has been shed on this problem in a study comparing rates of glucose disposal during lipid infusion in endurance-trained athletes and sedentary men ^12^. As expected, the lipid infusion resulted in a significant decrease in glucose disposal in the sedentary subjects. On the other hand, the glucose disposal rate and insulin sensitivity were less affected by lipid infusion in the endurance-trained athletes. Unlike sedentary controls, lipid infusion did not decrease rates of glycogen synthesis in the skeletal muscle of the endurance athletes. The latter finding suggests that glucose transport and glucose metabolism remained intact during lipid infusion in trained athletes. This conclusion was supported by the observation that GLUT4 concentration in muscle biopsy taken before and after lipid infusion was unchanged in the trained athletes while GLUT4 levels were significantly decreased by lipid infusion in the untrained participants. While a GLUT4 half-life of about 50 hours has been measured in cultured 3T3-L1 adipocytes, the direct measurement of GLUT4 half-life *in vivo* or in muscle has not been made. Therefore, the lipid-induced decline in GLUT4 protein content over a 6-hour period in this study suggests that increased turnover may be responsible for the decline in GLUT4. Importantly, this study provokes questions about the metabolic regulation of GLUT4 expression. Further work is required to understand the mechanisms that regulate GLUT4 protein synthesis and degradation in a physiologic setting.

It is well established that exercise is an important lifestyle intervention for enhancing both glucose disposal and insulin sensitivity. One of the key features of exercise is increased energy expenditure by working muscle, including the heart. Work by multiple laboratories demonstrated that glucose clearance in exercise-trained rodents was correlated with GLUT4 protein expression in skeletal muscle (reviewed in 13). Although GLUT4 protein is increased, it is likely that other adaptations in trained muscle may contribute to enhanced glucose clearance. For example, rats bred to have an intrinsically high aerobic capacity maintain high glucose disposal rates even when challenged with a high-fat, obesogenic diet ^14^. These rats were selected for their high-capacity running (HCR) and are compared to similar rats that were concurrently selected for their low-capacity running (LCR). Noticeably, the high-fat-fed HCR rats, even when maintained under sedentary conditions, showed increased glucose clearance and insulin sensitivity under euglycemic/hyperinsulinemic clamp conditions. Glucose clearance was largely accounted for by muscle glucose uptake and glycogen synthesis ^14^. In our lab, we have shown that transgenic overexpression of GLUT4 protein under the control of the human *GLUT4* gene promoter also enhanced glucose disposal under high-fat feeding conditions ^15^. In our model, glucose uptake by muscle and adipose tissue resulted in enhanced production of alanine, a potential gluconeogenic substrate for the liver. Pyruvate tolerance in these animals was enhanced, further supporting the notion that the glucose–alanine cycle was enhanced in the GLUT4 transgenic mice ^16^. In our model, the enhanced glucose–alanine cycle is perhaps serving as a futile, energy-consuming cycle that is increasing ATP turnover, at least in the liver. The HCR rats demonstrated a thermogenic phenotype and increased metabolic rate ^17^.

## Regulation of glucose transport by glucose metabolism

In the studies described above, glucose clearance is thought to be mediated largely by glucose transporter function. While it is clear that glucose transport relies on the number of cell surface glucose proteins, the rate of glucose uptake may be regulated by other factors, including intracellular glucose metabolism. This hypothesis was tested in a few ways. Fueger *et al*. showed that overexpression of hexokinase II increased muscle glucose uptake in chow-fed mice under hyperinsulinemic/euglycemic clamp conditions ^18^. This showed that, under normal conditions, an increased rate of production of glucose-6-phosphate enhanced insulin-mediated glucose uptake. The effect was lost when mice were fed a high-fat diet, suggesting that high-fat diet-induced inhibition of insulin-induced GLUT4 translocation could not be compensated by increased capacity for glucose phosphorylation. This notion was supported by our work showing that glucose uptake in the muscle of high-fat-fed mice was increased by transgenic overexpression of human GLUT4 protein ^16^.

The hypothesis that glucose transport can be regulated by glucose metabolism has been systematically tested *in vitro*. Tanner *et al*. measured glycolytic flux in immortalized baby mouse kidney (iBMK) cells that were systematically transduced with at least one human cDNA encoding each step of the glycolytic pathway beginning with the glucose transporters and ending with lactate transporters ^19^. This approach showed that glycolytic flux could be increased independently by overexpression of proteins regulating at four key steps. These steps include glucose transporters, hexokinase, PFK-1, PFKFB3, and lactate transporters. The output for this study was glycolytic flux rather than glucose transport *per se*. One might conclude that an increase in glycolytic flux would require an increase in glucose transport, unless increased glycolytic flux was diverting glucose-6-phosphate away from other pathways such as glycogen synthesis or the pentose phosphate pathway. While it is not likely that glycogen synthesis in the cultured cells is an active pathway, it is possible that the pentose phosphate pathway was active. Further experiments are required to determine if the four key steps that regulated glycolytic flux also regulate the rate of glucose transport. This is an intriguing possibility because understanding how glycolysis is regulated may provide important insight into the defects that underlie decreased glucose disposal in type 2 diabetes.

## Regulation of glucose uptake and metabolism in the heart

Among the insulin-sensitive tissues, the heart is unique because it has continual energy requirements and it never fatigues yet it lacks the capacity to store nutrients. Thus, the heart must be able to metabolize available circulating nutrients to meet these unyielding energetic demands. In healthy individuals, the heart primarily relies on fatty acids and, secondarily, glucose. However, like other insulin-sensitive tissues, GLUT4 is trafficked to the plasma membrane (sarcolemma) in response to insulin. Thus, after a meal, the cardiac uptake of glucose and glucose oxidation are increased.

It is well established that diabetes decreases the capacity of the heart to oxidize glucose and its reliance on fatty acid oxidation dramatically increases. This is referred to as metabolic inflexibility, and it is believed to be a significant component of diabetic cardiomyopathy. The underlying mechanisms of metabolic inflexibility are thus an area of intense research. Studies by William Stanley and others in the 1990’s demonstrated cardiac GLUT1 (the primary insulin-insensitive glucose transporter) and GLUT4 protein expression and glucose uptake are decreased in various animal models of diabetes ^20^. This is also true in humans who have type 2 diabetes and left ventricular dysfunction ^21^. However, the decrease in cardiac glucose uptake likely depends upon the duration of disease. In a more recent study, glucose uptake rates in several tissues were assessed by using 18F-FDG PET and MRI in conjunction with a hyperinsulinemic clamp. In this small cohort study performed on control, prediabetic, and type 2 diabetic patients, glucose uptake was decreased in the liver, skeletal muscle, and adipose of type 2 diabetics but unchanged in the heart. This suggests that cardiac glucose uptake is maintained, at least at rest and under the conditions of the hyperinsulinemic clamp, as compared to other tissues. Whether or not cardiac glucose uptake is maintained during increased workload, or how it proceeds with the progression of the disease, must be further explored.

Cardiac GLUT4 translocation and glucose uptake can also be stimulated in an insulin-independent manner in response to catecholamines. This is important for physiological and pathophysiological adaptations. For example, studies using a cardiac-specific GLUT4 knockout model demonstrated that GLUT4 is required for adaptations to hemodynamic stress resulting from swimming exercise or transverse aortic constriction ^22^. Older work also reported that there is increased myocardial glucose uptake during exercise and increased workload, but this is not necessarily mediated by increased myocardial GLUT4 content as seen in exercised skeletal muscle ^23, 24^. However, the effect of exercise on cardiac glucose uptake and glycolysis depends upon exercise duration. Chronic exercise training results in decreased cardiac glycolysis during training but is elevated approximately twofold 24 hours afterwards ^25^. This increase in glycolysis in the post-exercise phase is essential for cardiac growth. What is not clear, though, is how diabetes affects these adaptive processes in the heart. For example, if glucose uptake is increased in the heart by catecholamines, but downstream glycolysis is impaired, the beneficial effects may be compromised.

In addition to decreased GLUT4, additional mechanisms are also in place to limit basal cardiomyocyte glucose uptake by GLUT1 when confronted by hyperglycemia. Thioredoxin-interacting protein (TXNIP) is an inhibitor of the antioxidant enzyme thioredoxin, and its gene expression is highly responsive to glucose. A recent study by Myers *et al*. examined the role of TXNIP in the diabetic heart and used a proteomics approach to identify interacting partners ^26^. They found that TXNIP associated with GLUT1, and experiments performed in cell culture revealed overexpression of TXNIP decreased glucose uptake. Reciprocally, mouse embryonic fibroblasts isolated from TXNIP knockout mice displayed enhanced glucose uptake. This work suggests that unfettered glucose uptake via GLUT1 transporters may be limited in cardiomyocytes exposed to hyperglycemia by the increased expression of TXNIP.

As mentioned above, glucose transport is also affected by intracellular glucose metabolism. For example, in a mouse model that expresses a constitutively active form of PFK-2 (which increases the potent allosteric activator of PFK-1, fructose 2,6-bisphosphate) in the heart, there is an increased rate of glycolysis in the absence of insulin ^25^. This constitutive increase in glycolysis must be coupled with increased glucose uptake. In wild-type mice and in humans, cardiac PFK-2 is activated via phosphorylation by the insulin signaling cascade. This facilitates the metabolism of glucose, taken up by GLUT4, through glycolysis. In our own work, we have found that PFK-2 content is regulated by insulin signaling and that it is constitutively decreased in mouse models of diabetes ^27^. Regulation of PFK-2 content by insulin may serve as a mechanism to decrease cardiac glucose uptake and metabolism during fasting, but, in the context of diabetes, a chronic decrease in PFK-2 may contribute to metabolic inflexibility. Furthermore, changes in PFK-1 or PFK-2 activity may have effects on how glucose that is taken up is then metabolized. For example, the Hill group has recently shown in a metabolic tracer study in cardiomyocytes that the activity of PFK-1 is a key determinant in the fate of glucose entering into glycolysis or ancillary pathways, such as the pentose phosphate, hexosamine biosynthesis, and glycerolipid synthesis pathways ^28^. It has also been postulated that dysregulation of key glycolytic steps that affect PFK-1 or PFK-2 facilitate the production of glycogen, which abnormally accumulates in the diabetic heart (reviewed in 29).

In addition to the canonical GLUT1 and GLUT4 facilitative glucose transporters, cardiomyocytes also express other members of the GLUT family of proteins as well as members of the sodium glucose cotransporters (SGLTs) (reviewed in 30). Given that the majority of glucose is taken up by GLUT4, and secondarily by GLUT1, the physiological role of other glucose transporters still must be determined. GLUT8 has recently been implicated as having a role in insulin resistance-induced atrial fibrillation, but the mechanism is not clear ^31^. Like GLUT4, total GLUT8 protein expression is decreased in mice with obesity-induced insulin resistance ^31^. SGLT2 inhibitors have gained ample attention because, in addition to their ability to decrease blood glucose levels, they also have a positive impact on heart failure. SGLT2 is not expressed in the heart, and the positive effects of its inhibitors may be mediated by the systemic lowering of blood glucose levels or direct effects on cardiomyocytes via mechanisms that are still under investigation ^32^. A recent study has reported that the heart does express SGLT1 abundantly, though ^33^. SGLT1 transports glucose by an active transport mechanism using the Na ^+^ gradient. However, its functional role in the heart is independent of glucose transport and its importance to normal physiology and disease states, such as diabetes, must be further evaluated.

## Conclusions

The clearance of glucose from the blood plasma following a meal is important for overall metabolic health. Because the central nervous system is highly dependent on glucose for an energy source, multiple layers of regulation are in place to maintain a steady level of glucose in the blood plasma during both the fed and the fasted state. When this process breaks down, causing prolonged insulin resistance, the risk of developing type 2 diabetes and subsequent complications is heightened. Restoration of normal glucose disposal is the therapeutic goal for both insulin resistance and type 2 diabetes. It is likely that long-term success in reaching this therapeutic goal will rely on attacking the root cause of the glucose transport defect. Thus, if nutrient overload is at the heart of the development of insulin resistance and impaired glucose uptake, it stands to reason that matching energy input with energy output is the appropriate course to follow. Energy output might be enhanced by several approaches including increased physical activity, increased thermogenesis, or even enhancing the activity of futile metabolic cycles. Each approach leads to increased ATP turnover, helping to match energy output to energy input.
